# Circumcision: Pros and cons

**DOI:** 10.4103/0970-1591.60437

**Published:** 2010

**Authors:** Berk Burgu, Ozgu Aydogdu, Semih Tangal, Tarkan Soygur

**Affiliations:** Department of Pediatric Urology, Ankara University School of Medicine, Ankara, Turkey

**Keywords:** Circumcision, urinary tract infections, sexually transmitted diseases, penile cancer

## Abstract

Circumcision is possibly the most frequently performed elective surgical procedure in men. It can simply be described as the excision of the preputium. There have been several studies about the association between circumcision and urinary tract infections (UTI). Many studies have demonstrated that the frequency of UTI increase in uncircumcised males, especially in the first year of life. This review discusses the embryology of the preputium, epidemiology, indications, complications and benefits of circumcision, as well as operation and anesthesiology techniques. It especially examines the association between UTI and circumcision and the importance of circumcision in congenital urinary system anomalies. In addition, this review examines the associations between circumcision and sexually transmitted diseases, including HIV, and the protective role of circumcision on penile cancer.

## INTRODUCTION

Circumcision can be defined as the excision of the foreskin. The relationship between circumcision and urinary tract infections (UTI) is confusing due to the lack of standardization of definitions in the literature. This becomes more challenging to interpret when the association between circumcision and serious urinary tract congenital problems are to be analyzed.

## EMBRYOGENESIS AND HISTOLOGY OF PREPUTIUM

The skin of the penile shaft elongates during the 8^th^ week of gestation, and the prep utium begins to develop from this ectodermal extension. Initially, there is an adherence between the squamous epithelium of the glans penis and the inner surface of the preputium, which generally continues into the postpartum period. The prepuce can be retracted in only 4% of newborns, but this ratio rises to 90% at three years of age[1] and to 97% in uncircumcised men at 17 years of age. Retraction of the preputium involves splitting of the inner epithelium of the preputium from the glans. The separation generally occurs by desquamation of epithelial cells, which forms a caseous white structure called smegma. Nocturnal erections also play a role in the retraction of the preputium.

## EPIDEMIOLOGY

Circumcision is an ancient surgical procedure with a history of 15000 years, according to Egyptian mummies and wall reliefs, and has been performed for 5000 years in South Africa. The Middle East which presently contains the most crowded circumcised population has a slightly more recent history of 3000 years.

Twenty percent of all men worldwide are considered to be circumcised and this ratio may vary in different countries. For instance, the proportion of circumcised men is reported to be 48% in Canada, 24% in England, and 82% white men and 54% of African American men in the USA. Circumcision ratios may differ according to race and can also be performed for religious, cultural, and medical reasons as well as due to the request of the parents. Circumcision rate decreased from 90% in 1970 in the USA to 60% in 2000. Circumcision related expenses other than medical indications have not been paid since 1948 in England and since 1970 in Canada and Australia. Thirteen states of USA were added to these countries in 2004.

In previous decades, the American Academy of Pediatrics (AAP) declared different neonatal circumcision policies. In 1975 and 1977, the AAP advocated that there were no medical indications for routinely neonatal circumcision.[2] In 1989, the AAP argued that neonatal circumcision might have potential advantages besides the known disadvantages and risks.[2] In 1999, this argument was changed to, ‘Despite recent scientific proofs present the potential medical utilities of neonatal circumcision, these data are not sufficient for recommending routine circumcision.

Serious policies have been instituted recently against circumcision that depend on the idea that penile sensation diminishes nearly 50% after circumcision. Therefore, the decision should be left to the child when he gets older. The majority of anticircumcision movements refer to the procedure as, ‘genital mutilation.’ However, it has been shown that there is no difference between circumcised and uncircumcised men in their ability to sense extroceptive and tactile stimuli on the ventral and dorsal surfaces of the glans.[3] This definitely counters the idea of loss of penile sensation.

However, there are also situations where circumcision becomes inevitable. These include phymosis, paraphymosis, balanopostitis, balanitis xerotica obliterans, preputium cysts, penile lymph edema, ammonia dermatitis, and the use of clean intermittent catheterization. However, topical steroids can be used for some of these indications as alternative treatment.

Although there are different theories about the accurate time of circumcision, it is generally not recommended between the ages of two and six years (phallic phase) to avoid the development of castration anxiety.

## NEONATAL CIRCUMCISION TECHNIQUES

Besides classical surgical methods, three different circumcision clamps can be used in neonates: Gomco, Plastibell, and Mogen clamps. The more frequently used Gomco clamp provides a superior cosmetic appearance in the neonatal period. However, glans injuries are rarely seen with the Mogen clamp, and although the Plastibell clamp can be easily used,[4] it is not generally preferred due to the longer stay of the device on the neonate penis.

### Analgesia

The AAP recommends full analgesia for circumcision. Although there is a general belief that neonatal circumcision can be performed without analgesia, recent studies have shown the increase of pain and physiological stress in this period.[2] Different analgesic applications exist for neonatal circumcision, including topically applied lidocaine-prilocaine (EMLA) cream, dorsal penile blockage, subcutaneous ring blockage, sucrose-glucose, and acetaminophen.[2]

When compared to the placebo, crying time was shortened and the increase in heart rate was lower in children who were circumcised under EMLA.[5] However, although the analgesic effect of EMLA is enough for removing the adhesions and for the placement of the clamp, it is not sufficient for serious pain arising from removal of the foreskin. The most common side effect of EMLA is methemoglobinemia which may develop due to the metabolism of prilocaine.[5]

Dorsal penile blockage is another effective technique to depress the physiological and behavioral responses related to circumcision. The maximal effect generally begins after 60 minutes[6] and the crying time diminishes by 50% with dorsal penile blockage; minimal increase is seen in the heart rate in 76% of the children.

Studies have shown that ring blockage is more effective than EMLA and dorsal penile blockage, and has a longer duration.[5]

Although it has been shown that analgesic modes such as the oral administration of sucrose, glucose, or parenteral acetaminophen are more effective than placebo, it is widely accepted that they are not sufficient for the circumcision procedure.[2]

### Complications of neonatal circumcision

Common complications of circumcision include hemorrhage (35%), wound infection (10%), meatitis (8-20%), and UTI (2%) respectively. Opening of the wound, insufficient removal of the foreskin, skin bridges and inclusion cysts, amputation of the glans penis, sepsis, phrenulum breve, and buried penis are rarely seen.

## CIRCUMCISION AND URINARY TRACT INFECTIONS

Several studies have examined the association between circumcision and UTI.[7–9] Only a few (2.5%) infants who were younger than 60 days of age and presented with fever, tended to be diagnosed with UTI.[10] It has been shown that the risk for UTI increased, especially during the first year of life, in uncircumcised infants.[10,11] In a study published in 1998 on 58000 children from Canada, who were less than a year of age and diagnosed with UTI, the risk for UTI was found to be 1.88/1000 in circumcised children and 7.02/1000 in uncircumcised ones.[12] Another study showed that the risk for UTI in similar subjects was reported to be 1.4 and 0.19% in uncircumcised and circumcised children respectively.[13] The incidence of UTI was shown to be 7-14/1000 and 1-2/1000 in uncircumcised and circumcised children respectively in a study organized by the AAP in 1998 on children during their first year of life.

Similarly, the rate of hospitalization among children with UTI was also higher, being 1/140 and 1/530 in uncircumcised and circumcised children respectively.[11]

Despite the existence of large amounts of data on circumcision and UTI, the lack of standardization in terms of urine collection and definitions, minimizes the value of these data. None of the data mentioned above were obtained by standardized urine collection methods or in similar patient groups, which makes their interpretation more complicated. For instance, the risk for UTI has been found to increase in premature newborns but decrease by three fold in children who are breastfed. The method of urine collection has also been reported to affect infection rates.[7] There are three different methods for urine collection in children: Suprapubic aspiration, catheterization, and the use of a bag. Suprapubic aspiration is considered to be the gold standard,[12,13] but the only study that reported this method as the standardized method of urine collection, reported a risk for UTI that was increased by ten fold in uncircumcised children.[9]

There is a reasonable biological explanation for the increase in the incidence of UTI in uncircumcised children. Increased periurethral bacterial colonization is a potential risk factor.[10] Uropathogenic colonization occurs to a greater extent around the meatus in uncircumcised children compared to circumcised ones in the first six months of life. After six months, the risk of colonization decreases for both groups.[8] However, an experimental study concluded that even if uropathogens could attach to the mucosal surface of the preputium, they could not proliferate on the keratinized surface.[14] Although it has been shown that the risk for UTI increases 4-10 fold in uncircumcised children, it is still not sufficient to recommend routine circumcision in the first year of life.

Results are even more confusing when the association between serious urinary system problems and circumcision is examined. A recent study examined the association between circumcision and vesico-ureteral reflux (VUR), the posterior urethral valve, and recurrent urinary tract infections. Fifty-nine uncircumcised VUR patients who were under antibiotic prophylaxis were examined for the effect of routine prophylaxis on foreskin bacterial colonization. Periuretheral culture samples were compared with samples of 36 healthy control patients who were not receiving antibiotics. Uropathogens were isolated from 37% of the patients in the first group and 28% of the subjects in the control group.[15] Thus, it appears that antibiotic prophylaxis is not sufficient to decrease bacterial colonization on the preputium and circumcision is essential in such patients even when they are under prophylaxis. Possible reasons could be the presence of a moist environment that is suitable for colonization and insufficient transition of the antibiotics to the inner part of the preputium.

However, there are also reports of controversial studies that are against routine circumcision in such patients. For example, UTI rates were found to be similar in a study on 28 children who had previously undergone successful antireflux surgery with circumcision and 29 children in whom only antireflux procedures were performed. Hence, the study concluded that circumcision should not be recommended routinely in such patients.[16] Shortly after the aforementioned report was published, a comment was published advocating just the opposite in 2004. Eighteen VUR patients were followed by circumcision alone (without prophylaxis or reflux correction), and whereas UTI was not seen in follow-up in 12 of these patients, it was observed repeatedly in six patients. In other words, circumcision was highly protective alone for UTI. 6 patients who had recurrent UTI were immune suppressed due to chronic renal failure.[17]

## CIRCUMCISION—SEXUALLY TRANSMITTED DISEASES

The association between circumcision and sexually transmitted diseases including HIV, has been found to be quite complicated. Previous studies have demonstrated that there is lower risk for syphilis in circumcised patients as compared to uncircumcised ones. Langerhans cells that exist on the inner surface of the preputium, are close to the surface because of the thin structure of the keratin layer and these cells are critical for some infections. In uncircumcised men, the presence of the thin, keratinized mucosal layer may allow the passage of viral pathogens to the lymphoid cells (Langerhans cells). Removal of the foreskin will block this potential port. For this reason, the World Health Organization has made a great effort for circumcision to be widely performed in Africa.

## CIRCUMCISION—PENILE CANCER

Penile cancer is rarely seen and has an incidence of 0.9-1/100000 in the USA.[18] Previous studies have demonstrated a negative correlation between circumcision and penile cancer.[19] The most important risk factor for penile cancer is phymosis, which explains the fact that neonatal circumcision is more protective against penile cancer compared to circumcision performed at older ages. Other risk factors include smoking, genital warts, and multiple sexual partners.

## CONCLUSION

Neonatal circumcision has been shown to have a protective effect against UTI. Circumcision decreases the incidence of febrile UTI, especially in the first six months of life. However, there are not enough data advocating routine neonatal circumcision. Clinical benefits of circumcision are clearer in urinary system pathologies, including VUR, neonatal hydronephrosis, and the posterior urethral valve.
